# Planned or emergent? An evaluation of a Master’s in Health Professions Education programme

**DOI:** 10.1186/s12909-022-03319-5

**Published:** 2022-04-04

**Authors:** Elize Archer, Susan Camille van Schalkwyk, Mariette Volschenk, Anna Maria Susanna Schmutz

**Affiliations:** Centre for Health Professions Education, Faculty of Medicine and Health Sciences, Stellenbosch University, Cape Town, South Africa

**Keywords:** Programme renewal, Masters in health professions education, Programme evaluation, Supporting relationships

## Abstract

**Background:**

Programme developers have the responsibility of ongoing programme renewal and evaluation to ensure that curricula remain responsive to rapidly changing educational and healthcare contexts. In reporting on programmes, significant emphasis is often placed on content and outcomes of Master’s in Health Professions Education (MHPE) programmes. However, less emphasis has been placed on meaningful evaluation of all aspects of these programmes, particularly from a student perspective including what worked and what needs to be enhanced, as well as any emergent or unplanned factors. As the number of established MHPE programmes increases, so does the need for evaluation models that consider programme complexity. In this article we consider a MHPE programme against a model that provided scope for going beyond ‘did it work?’ Our intention was to determine whether the renewed MPhil in HPE programme was implemented as planned, and to which extent it achieved the planned outcomes.

**Methods:**

This programme evaluation was conducted in an interpretive paradigm. We collected qualitative data at two points. Firstly, at the start of students’ first-year with voluntary participation in focus groups and secondly, a year later with voluntary participation in individual interviews. Two members of the research team performed the initial thematic analysis of both the focus group interviews and the individual interviews. Thereafter, the full author team worked collaboratively discussing the themes until we reached consensus, looking specifically to identify any “emergent” factors.

**Results:**

We identified three themes in the student data related to the process of implementing the new programme and the outcomes from it, including those aspects that could be regarded as emergent or unplanned: balancing work, personal lives and studies; managing the hybrid learning approach; and the scholarly journey.

**Conclusions:**

While many of the outcomes of the renewed programme were met, not all manifested as had been planned. The experience of the programme differed from one student to the next such that at the end of the two years they were at different points in their scholarly journeys. We realised that although we sought to be pedagogically sound in the process of curriculum renewal, we did not take into account the complex matrix of influences that sit outside the formal curriculum. Future renewal activities should intentionally and sensitively consider those factors, both planned and emergent, that influence a student’s journey towards becoming a scholarly teacher and teaching scholar.

**Supplementary Information:**

The online version contains supplementary material available at 10.1186/s12909-022-03319-5.

## Introduction and background

Over the last two decades, global trends towards the professionalization of health professions education (HPE) have led to a marked increase in the offering and popularity of Master’s degree programmes in the field [1–3]. Although there are variations in design and delivery, these programmes typically aim to develop educational experts, scholars and leaders in the health professions [2]. As the numbers of established MHPE programmes continue to grow, programme developers are charged with the responsibility of ongoing programme evaluation and renewal to ensure that curricula remain responsive to rapidly changing educational and healthcare contexts [2, 4, 5]. Accordingly, the World Federation of Medical Education (WFME) published *Global Standards for Quality Improvement in Master’s Degrees in Medical and Health Professions Education* [6] to guide standard setting and quality assurance, enhance excellence, and foster best practice development at MHPE programme level [5]. The document provides a comprehensive set of ‘criteria and mechanisms’ to inform evaluation, but it does not necessarily offer insight into how to conduct such evaluation in a scholarly and rigorous manner. Similarly, while there is significant emphasis on the content and outcomes of MHPE programmes in the literature [1, 5] there is less reference to theories and methodologies that may guide the meaningful evaluation of these programmes.

It has further been argued that often educational programme evaluation models tend to follow linear approaches that are influenced by reductionist theory and focus on associations between individual programme components and outcomes [7, 8]. Instead, the complex and dynamic nature of HPE necessitates evaluation models that consider programme complexity, focus on change related to outcomes (both planned and unplanned), emphasise the importance of context, and allow for the development of emergent theory [7–9]. It is here that complexity theory can provide a useful lens when applying an evaluation model that allows us to embrace the uncertainty and ambiguity characteristic of educational programmes. Frye and Hemmer (291) assert that complexity theory “invites educators to cease relying on overly simple models to explain or understand educational events”[9].

This article describes a response to this broader remit with a specific focus on the student voice. It documents the evaluation of a MPHE programme, considered against a model that provided scope for going beyond ‘did it work?’ [8], to enable the ‘broadening’ and ‘advancing’ of theoretical positions [10], beyond the context of the programme itself.

### The Master’s in Philosophy in Health Professions Education (MPhil in HPE)

The Centre for Health Professions Education (CHPE) at Stellenbosch University’s (SU) Faculty of Medicine and Health Sciences (FMHS), South Africa, has been offering an MPhil in HPE since 2008. As an established MPHE programme with 91 graduates, and one of only four in Sub-Saharan Africa, this two-year long modular programme is offered in a hybrid learning mode. Apart from a compulsory residential contact week per study year, and virtual meetings during each module, all learning is facilitated via an online platform. Online support is provided by a group of dedicated tutors, throughout the modules, and learning is deliberately integrated and scaffolded. Students in the programme represent different health professions and generally come from within the institution or from elsewhere in South and Sub-Saharan Africa. Although many are specialists in their respective fields, they are new to the field of HPE. Most work fulltime in challenging resource constrained environments and often have to balance their studies with a wide range of personal and professional commitments.

**Fig. 1:**
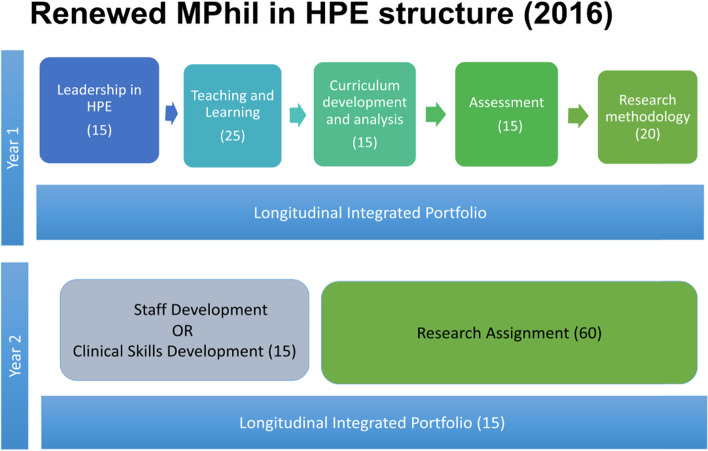
Each block represents a different module and includes the module name and the allocated number of credits in brackets. Students complete all course work modules, before embarking on the Research Assignment. Total credits for the programme = 180 (1 credit equals 10 notional learning hours)

In 2016, following on a two-year, full-scale programme renewal process, a new curriculum was implemented (Fig. 1).

Apart from revisiting core content, the renewal process intentionally identified key educational principles, or so-called “golden threads”, that would knit the modules of the renewed programme together to facilitate an integrated curriculum experience. These golden threads were premised on the principles of two established adult learning theories namely transformative learning theory [11, 12] and self-regulated learning [13], and included inter-professional practice, social accountability, an evidence-based approach, and the development of graduate attributes such as reflective practice, role modelling, as well as the ability to act as change agents in an educational context. In this context, transformative learning theory was understood as learning that occurs when students are confronted to critically consider their ‘ways of being’ in the world, challenging fixed ideas and dominant thinking with a view to becoming more inclusive and able to change. This is facilitated when the adult learner is actively engaged in a process of reviewing internally generated feedback on their learning, thereby taking responsibility for regulating such learning [14].

Accordingly, an integrated electronic portfolio (e-Portfolio) module was introduced to support the longitudinal development of students’ personal teaching philosophies through engagement with mentoring, feedback from multiple sources (e.g. 360 degree assessment on leadership skills, feedback on module assignments, feedback on reflective portfolio entries), self-generated feedback (where students themselves approach lecturers and mentors for input), regular critical reflection, and professional development planning. This module was seen as a vehicle that would allow students to recognise the interconnectedness between the various modules and to think in new and transformed ways.

The overall programme outcomes are depicted in Table 1.

**Table 1:** The four programme outcomes planned for the renewed curriculum

Our Master’s in HPE programme aims to achieve the following in our graduates:
1. to promote excellence with respect to education, research and community interaction in the field of HPE on the African continent;
2. to facilitate research and academic reflection in order to make a scholarly contribution to the body of knowledge in the field of HPE;
3. to ensure a rich learning environment fostered through multi-disciplinary and international perspectives and
4. to develop HPE leaders who can contribute to the advancement of evidence based practice in Higher Education in Health Sciences in the interest of enhancing the quality of health care in Africa

The above outcomes are aligned with the *university’s* teaching and learning policy, that describes the progression of the teaching role from reflective practitioners, to scholarly teachers, to becoming teaching scholars and lastly, leaderly teaching scholars [15]. In the policy, reflective practitioners are those who engage with their teaching practice and professional growth in a deliberate and critical manner. Scholarly teachers move beyond personal reflection, drawing on educational literature to reflect on their teaching practice and professional growth, while incorporating observation and peer review of their teaching. Teaching scholars engage in systematic enquiry of their teaching practice, contribute to the body of teaching and learning knowledge through sharing their findings publicly. Ultimately, leaderly teaching scholars provide educational leadership at institutional, national and international level, in addition to contributing to the body of teaching and learning knowledge through publication [15, 16].

### An analytical frame

**Fig. 2:**
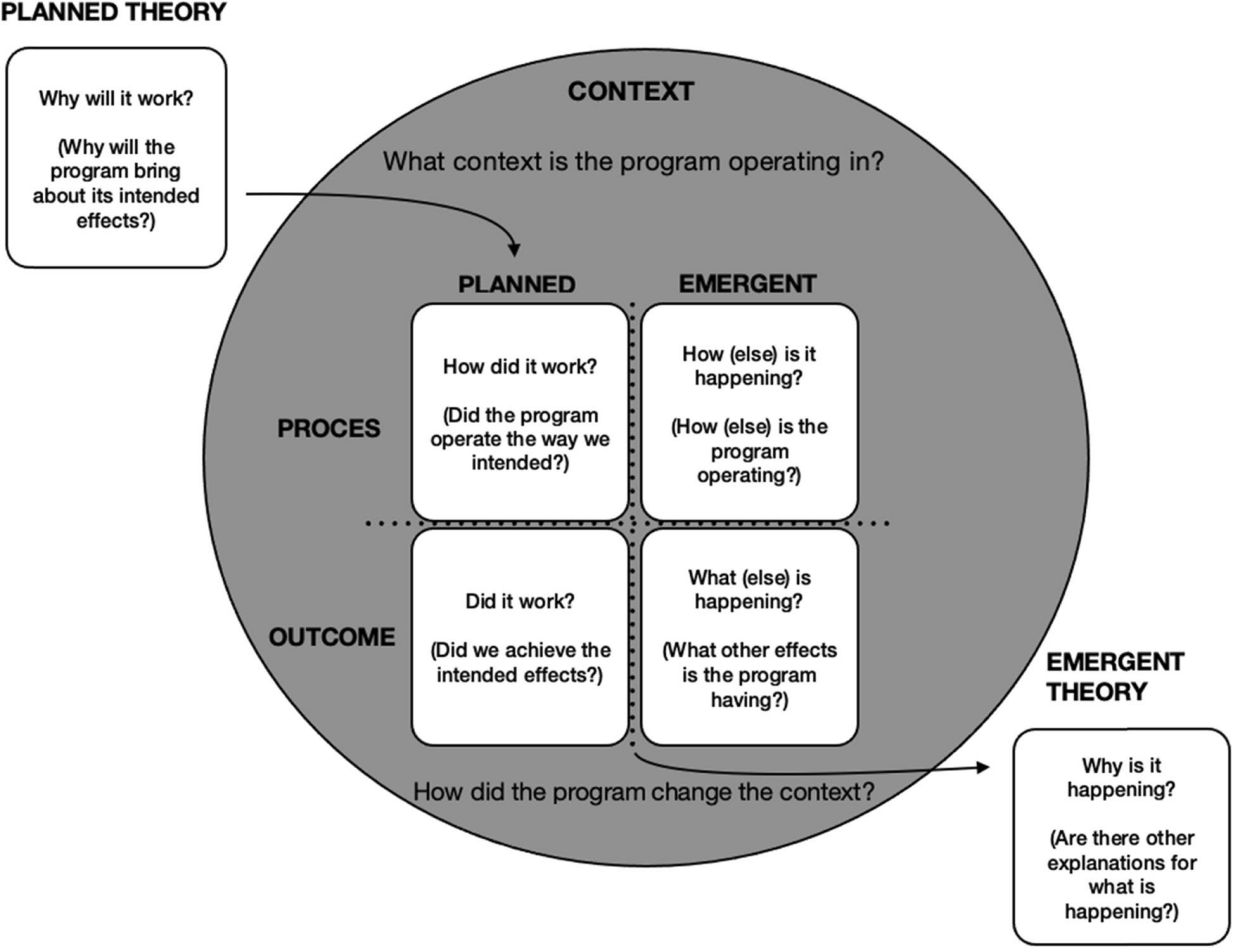
Haji, Morin and Parker’s Framework for Program Evaluation (2013) (Permission from the authors received)

In the interest of being accountable to both the institution and our students [6] we as the team largely responsible for designing and operationalising the new curriculum, embarked on a study during the first two years of implementation. Our initial intention was to determine (1) whether the renewed MPhil in HPE programme had been implemented as planned, and (2) the extent to which it had achieved its planned outcomes [17]. However, given our concerted efforts to adopt a process of programme renewal that was informed by current evidence and sound educational principles, we sought to go beyond purely determining whether we had achieved what we set out to achieve and also identify any emergent outcomes of the programme. While several evaluation models are available in the literature [9], we believed that the seven element framework proposed by Haji and colleagues [8] (Fig. 2) would afford us an analytical framework that would fit our purpose. This framework not only considers the planned theory that underpins the planned curriculum processes needed to achieve the planned outcomes, as described above, but also takes into account those processes and outcomes that emerge during implementation. These emergent properties speak to what *else* happened over and above what was predicted. The four positions are set within the context within which the programme operates, as a means to inform emergent theory development [8]. This article documents the outcomes of our evaluation considered in light of this model.

## Methodology

This longitudinal evaluative research project was conducted in an interpretive paradigm [17]. We collected qualitative data at two points in order to understand the experiences and perspectives of students regarding the processes and outcomes of the programme. Informed consent was provided by all the participants in the study and convenience sampling was utilised for selection of study participants.

The first point of data collection was at the start of their first year. We approached the 2017 student cohort (*n* = 13), informed them of the purpose of the study and invited their voluntary participation in one of two focus groups. These were scheduled during a lunch break (13:00 – 14:00) on consecutive days during their residential period. Twelve students (6 per group) participated in the focus groups that were facilitated by an experienced interviewer, not linked to the programme. One student was unavailable. The interviewer asked students to reflect on their learning experiences during their first-year in the programme. The prompts (see Additional file 1: Appendix 1) were prepared to facilitate discussion around the first-year modules and learning experiences as well as to establish factors that enhanced or constrained student learning. The interviews were recorded and transcribed verbatim by an independent transcriber.

The second point of data collection occurred a year later in 2018. The same cohort of students, now in their second year of study, were invited via email to participate in individual interviews. Seven students agreed to participate; the rest were either unavailable or had not yet completed all the coursework related to the programme. Our intention with the interviews was to explore in greater depth the issues previously raised in the focus groups regarding their learning experience and to get a sense of possible shifts in the students’ perspectives now that they had completed either the entire programme, or at least the coursework component thereof. Logistics informed the decision to conduct all individual interviews with students either telephonically or via SKYPE. A set of open-ended questions was used to prompt students’ perceptions and reflections on their expectations and experiences. The interview schedules (Additional file 2: Appendix 2) for both the focus groups and individual interviews were informed by the principles of Transformative learning theory [11, 12]and were based on the overall programme outcomes (Table 1) and the educational principles that the programme is built upon. The interviews were audio recorded with informed consent and transcribed verbatim, before sending the transcripts to the participants for member checking with a request to clarify, modify, or express disagreement. Only one participant replied providing an additional comment to clarify a statement in the transcript.

Two members of the research team performed the initial thematic analysis of both the focus group interviews and the individual interviews. Following on Saldaña, the transcripts were read several times, codes were identified, and a codebook was developed. Codes were then clustered into themes [18]. Thereafter, the full author team worked collaboratively discussing the themes until they reached consensus [18], looking specifically to identify any factors that could be regarded as ‘emergent’ [8].

## Findings

We identified three themes in the student data related to the process of implementing the new programme and the outcomes that resulted from it: balancing work, personal lives and studies; managing the hybrid learning approach; and the scholarly journey. For each of these themes there were sub-themes. The responses from the interviews are reported as follows: Year I and focus group number (YIFG1 or YIFG2); Year II and participant number (e.g. YIIP1).

### Theme 1: Balancing work, personal lives and studies

The issue most raised by the respondents had little to do with the programme itself, and much more with how the programme influenced their day-to-day lives. All of the students were working fulltime and they described difficulties in having to balance work, personal circumstances and studies, and that this was often contrary to, or at least far more of a challenge than, what they had expected. There was also a sense that the challenges they encountered were not given due consideration by their tutors.

*I think I personally knew that it was going to be hectic, knowing that you’re working, family, and studying. So by no means did I expect to be given things, or even be – what’s the word – let things be easy. Oh shame, she’s a working person, let things be a little bit easy on you. But we need a little bit more consideration with the fact that we are full time employees, and yet we are expected to be full time students (YIFG1).*

Balancing work, personal life and study was further complicated by what the students experienced as variance across the different modules, and by a sense of compartmentalisation – studies versus ‘*the other part of our life …*’ (YIFG2) – on the one hand, and a sense that being an adult learner had added to their many already established roles. The quote below reiterates what the previous participant highlighted, namely that students experienced the various modules quite differently and also that they battled to balance their personal life with their study responsibilities.

*It’s a mix of different experiences, different, you know. There are dramatic times, there are exhilarating times, there are times where you experience growth, there are times that you feel crushed, there are ego bashing times, and there are ego lifting times. So you know, it’s a mixture of everything, and it varies with the module and what one is going through in the other part of our life at that time. So, I mean as in adult learning, you are not only doing this course, you’re a father or a mother, you're a husband, you’re an employee, you’re part of society, you’ve got obligations to society, you are very many things (YIFG2)*.

The pace of the programme, with modules following quite quickly after one another also presented challenges for students trying to navigate a way through the many demands being made on them.

*You do put a lot of your other responsibilities on hold, so it’s almost like you just need that time to recuperate and just try to get everything settled, just to organise your life again. So I just felt that I just needed maybe those few days in between (YIFG1).*

*The one module was finished, your assignment went in with nothing, boom, eight o'clock the next morning, the next module began, and the lecturers were saying hello, where are you [laughs]. Why aren’t you engaging [laughter]? Goodness gracious! Actually, give me a break, we’re still recovering (YIFG1).*

By the end of year two, the students’ reflections still emphasised how hard it had been for them to get to their studies while working full-time. They specifically highlighted how the tight structuring of the programme influenced the quality of their learning, not allowing time for reflection. *The modules were so close to each other and at the end of a module; you just never had the time to sit back and take a break and reflect properly (YIIP4).*

### Theme 2: Managing the blended learning approach

Students, particularly the first-year group, also expressed difficulty engaging with the university’s online (SUNLearn) platform as a learning space. For some it was the platform itself that caused frustration, while others found it difficult to get used to a blended learning approach. Feelings of isolation, and experiencing stress in trying to maintain the pace, again related to the programme’s structure, were mentioned.

*What was challenging for me at the beginning was to get to grips with SUNLearn. It was my first experience with SUNLearn, and we almost left here, after the contact week, and I had no understanding of SUNLearn, and that was my only contact, or my only means of communication with the rest of the group. So I kind of felt isolated because I didn't know how to get onto it, how to load my things (YIFG1).*

On the other hand, one student specifically mentioned the benefits of the online platform, including the affordances it offered for social learning.

*I think the way that the course run, the way that it was structured logistically - it’s easy, it was pleasant to do, it was engaging. There was always opportunity to speak to the people who are involved in the teaching of the module, but also there is always opportunity to engage with your peers. And I think as a student if you can have engagement from your peers, it’s sort of a journey that you can be on together, even though majority of it is facilitated by distance learning, I think that didn’t change or take away any value of learning with a group of people other than feeling like you’re on this all by yourself (YIIP2).*

Although the curriculum had been intentionally designed to foster coherence across the entire programme, this was not necessarily the students’ experience during the year-long online engagements with several noting how their experience differed from one module to the next.

… because I think that I just felt very different from one module to another depending on who was leading the module and how often I interacted online (YIIP3).

There were, however, factors that served to ameliorate this concern such as the longitudinal e-portfolio, which was described as a tool that facilitated coherence across the programme.

… So if we look at each of the modules – these modules occurred in silos, if you want to say it like that—but the e-portfolio allowed you the opportunity to marry it together (YIIP4).

Interestingly, in spite of the focus on engagement within the online forums, the WhatsApp group that the students created for themselves was foregrounded as a key source of support. WhatsApp is a popular instant message mobile application for Android and other phones.

*So, I think one of the wonderful things is that we have all been on a WhatsApp group throughout the time period, and so within the students I think that’s been very supportive, and we have been able to ask a lot of questions amongst ourselves, and feel like you are part of a class, even though you are doing it online and don't see each other. So I think that was really excellent (YIFG2).*

### Theme three: The scholarly journey

*It’s sometimes crushing, it does crush the ego, it does have its advantages because it sometimes makes me realise oh, alright, you’re not as good as you thought, right (YIFG1)?*

*I found the writing centre beneficial. They didn't comment much on the content per se, but about the writing, because it was a mind-shift for me to write what we needed to, and that I found useful (YIFG2).*

*Well, my personal experience of mentor was a pleasant one. I had good communication. I always felt I could, she was very approachable, I could always approach her when I needed help (YIFG1).*

Evident in the responses from the year two interviews was a sense of growth. Academic support that had been built into the programme such as each student having a mentor (also mentioned in the first year) and structured guidance from the writing centre, were described as enabling in this regard. Students further described how their learning had been informed by observing and interacting with the facilitators, and how this had influenced their own practice. *I learned a lot from observing and interacting with the facilitators. Those things that I now also try to incorporate or the way that they supported learning…To not only learn of these things, but to see in practice (YIIP1).* Here it is interesting to note that while consciously focussing on using different teaching methodologies and group techniques to facilitate learning had been planned, our ‘way of doing’ was also a source of (unplanned) learning.

Most evident, however, was the clear sense of a shift in their thinking and understanding around teaching and learning, and how that shift scripted their journey towards becoming a scholarly teacher, becoming more critical and reflective, seeking to influence the field.

*…when I entered the programme I thought it was about improving myself, where I now really view myself as …somebody who should be critical in improving education and teaching and learning at the University and particularly also in my programme (YIIP1).*

*I feel like I have the confidence and the knowledge to voice that and to actually have an opinion and have that opinion that I am able to sufficiently back with evidence and things that I learned on the MPhil (YIIP2).*

Confidence in terms of taking on a more scholarly or academic identity, however, varied across the group, with some positioning themselves for further studies, while others feeling that they still had some way to go.

*I am trying right now to plan more research based on educational scholarship than previously understanding some of the basics of that because of the MPhil and that is something that I want to pursue more over the next couple of years and I am also thinking about the possibility of a PhD. So, I do think it changed kind of the way I looked at things from that perspective (YIIP3).*

*I think it’s [the programme] been really good, it has been a great experience, but still as a scholar and a writer, I’d say definitely has this little lot but definitely still baby stages - I think. I’ve been able to put a lot of it into practice – what I’ve learned into practice. But with regards to actually being an academic, I think definitely still baby stages (YIIP7).*

This growth and new insight was ascribed to engaging with critical reflection; intentionally responding to feedback; and collaboratively engaging with health professionals from different disciplines in the many programme discussion forums.

*the self-reflection and the personal development plans that you had to do and asking your peers and people that you directly work with, to give feedback to you – even though it wasn’t always nice to read the feedback – I think the value of the information that came from that and you having to reflect and come up with how am I going to become a better educator and I certainly became a better person personally from all of that (YIIP2)*

*…and I really enjoyed being with people of different professions and health educations and not just nursing. So that was a great experience and I think I have, I think one of the biggest things were the sort of growing, and growing in confidence (YIIP7).*

Growth was characterised by having confidence in their ability to fulfil a role as educational leader and advocate, particularly in their own institutions.

*So when I look at the MPhil holistically, I realise that it wanted me to become an agent of change and that is why I said it surpassed my expectations, because it made me aware of the need of myself to become an agent of change. So that’s when I look at initial expectations, it was really selfish expectations. It was to develop myself, to better myself and to [inaudible] others to propel myself in academia, but when I walked out of the MPhil. I realised that those initial expectations were selfish and now I want to take this knowledge that I have or the experience that I have and the exposure that I have and try to empower as many colleagues as possible so that in that way we can better instruct our new students (YIIP4).*

In sum, the findings demonstrate a shift in the responses related to students’ personal and scholarly growth from year one to year two. While many students seemed to feel out of their comfort zone during year one, this appeared to change over time. Furthermore, while we intentionally looked to identify emergent processes and outcomes, over and above the planned processes and outcomes, the lines between these were blurred, suggesting a continuum of understandings rather than a dichotomy as might be assumed from Haji et al.’s model [8]. Finally, present in all of the themes was the idea of variance, emphasising the unique nature of each student’s experience. These ideas are explored further in the section that follows.

## Discussion

This study set out to go beyond determining whether the renewed MPhil in HPE programme had been implemented as planned, and the extent to which the planned outcomes had been achieved. What can be learnt from our analysis? Firstly, that curriculum developers and those responsible for facilitating learning should not underestimate the unique nature of each individual student’s journey. Artino, Hemmer and Durning [14] describe self-regulated learning as a ‘multidimensional construct’ that engages the ‘whole’ person – behaviourally, cognitively and emotionally. We realised that despite careful planning, and even if the implementation occurs according to that planning, the histories, contexts and needs that people bring with them to a new learning situation are complex, and the experiences they have of the programme will play out differently for different individuals. This can result in a tension between trying to take responsibility for one’s learning, while seeking to maintain some semblance of work-life balance. This holds important implications for curriculum developers involved in postgraduate learning, especially when those postgraduate learners are adults who are studying part time. Time for maintaining equilibrium, on the one hand, while allowing for the critical reflection that was expected, on the other, becomes key to providing a meaningful learning experience.

The complexities discussed here foreground the importance of both general and targeted needs assessment prior to curriculum design and implementation [19]. In hindsight we acknowledge that a thorough needs assessment of prospective students prior to programme implementation may have better prepared us as curriculum designers for the degree to which various personal and professional circumstances and responsibilities would dictate the nature of programme participation. This could perhaps have allowed us to put measures in place to negate these challenges more effectively. Although we suspected that some of these issues would be highlighted by the study findings, we did not anticipate the intensity with which students in our study experienced their individual challenges. In that regard, the research findings proved very insightful and will inform our planning going forward.

Another focus relates to the role of transformative learning as an underpinning theoretical principle which informed the programme’s design, including the focus on becoming reflective practitioners and agents of change. Mezirow describes how, in the context of transformative learning, students can be confronted during the course of their learning by a ‘disorienting dilemma’, one that leads them to question previously held beliefs and positions [11]. It could be argued that students were confronted in this way, but the source of the dilemma was not only embedded in the content of the different modules, but also in their lived experience of the programme itself. This type of complexity requires our further attention. A recent critical review of transformative learning [20] has questioned the ethics of intentionally introducing disruption in the classroom, challenging students to confront their dominant perspectives and long-held beliefs – a key dimension of transformative learning theory – and called for sensitivity on the part of educators in this regard. Our analysis highlights the need for such awareness, reminding us that the learning experience is always framed within a broader context [21]. Yacek’s work further resonates with what we found in this study as he emphasises the importance of ‘community’, as seen in the students’ reliance on the WhatsApp group, that extends ‘beyond the classroom’ [20]. This not only highlights the potential to foster peer support at postgraduate level, but also suggests a role for us as facilitators and supervisors, to draw our graduating students into our disciplinary community with a view to providing ‘a continuous undergirding for the moments of discontinuity … that transformation entails’ [20].

We were further struck by how the students’ lived experiences of the two years were characterised by uncertainty rather than an awareness that what they were struggling at the time in terms of new ideas, different discourses, which would all come together in the end. Previous work has explored the postgraduate journey as one characterised by uncertainty and lack of confidence [22] and we were reminded that while we had structured the programme as a journey across the two years, the implication of the modular structure was that students instead experienced it as a series of short sprints interspersed with ‘high stakes’ assignments. We realised the importance of making students aware of the over-arching picture, and regularly bringing them back to the overall ‘route map’, the golden threads, and the principles that informed the programme architecture in the first place.

The diversity across the students’ experiences within the programme was also seen in the extent to which they had moved towards recognising ‘excellence with respect to education, research and community interaction in the field of HPE’ (programme outcome #1) and ‘making a scholarly contribution to the body of knowledge in the field of HPE’ (programme outcome #2). Van Schalkwyk, Cilliers, Adendorff, Cattell and Herman [23] have mapped a scholarly journey for teachers moving along the continuum from reflection to scholarship – the sort of scholarship envisaged by these programme outcomes. While we were excited to see how some students described being confident and having travelled some way along their scholarly journeys by the end of the end of the programme, we also acknowledged how others described their progress as ‘baby steps’. It was evident that this planned outcome had not yet been met by our students at the end of year two.

As curriculum developers we have already implemented some actions to support the students more, of which the focus on individual mentoring of students is one that we see as very important. Students are encouraged to check in with their mentors regularly and seek support as they require. From an overall programme coordination point of view, we also provide opportunities for online discussions about twice a year so that students can be reminded of the “bigger picture” of the programme and not feel that they are not alone in this learning journey that they have embarked on.

A possible limitation of the study, is that it only included student voices and no other stakeholders were engaged. We included just one cohort of students, and only four of the respondents had completed, submitted and passed the final research component of the programme at the time of the research. We realise that the exclusion of other students’ experiences limited the data that we could analyse for the study. It is likely that further follow up a year later would have seen more having progressed on their scholarly journeys. Nevertheless, we believe that in fostering ‘a rich learning environment through multi-disciplinary and international perspectives’ (programme outcome #3), the Master’s programme does indeed set the graduating student on a course toward scholarship. While not describing scholarly activities per se, many of the students described feeling confident to return to their work environments and take up a leading role in teaching and learning (thus programme outcome #4). A further limitation of focussing on a single cohort of students, the group that was first exposed to our renewed curriculum, is that we cannot be sure that this group’s experiences was any different from previous or later groups. Further longitudinal research is needed to track the graduates of the different cohorts over time.

## Conclusion

We contend that overall, the programme outcomes for the renewed MPhil in HPE programme were met to a greater or lesser degree, but not necessarily in the way that had been envisaged. The extent to which the entire experience of being an MPhil student influenced their individual and unique scholarly journeys, was unplanned. During the process of curriculum development, we had focussed our attention on being pedagogically sound, our work informed by recognised adult learning theories. Accordingly, we had expected that our students, as experienced professionals, would adopt the principles of self-regulated learning with relative ease. We had further envisaged that the students would embrace the tenets of transformative learning through conscious and critical reflection. In the implementation, however, we were reminded that there is a matrix of influences sitting outside the formal curriculum, directly influencing the way in which the programme manifests for each student. The health professionals in our study, who had again taken on the role of student, now had to grapple with finding a balance between their work, personal lives and their studies. We saw that technical expertise in the learning online environment is a necessary, but not sufficient condition and that there is a need for community when studying remotely. Finally, we recognised that implicit in becoming a master in health professions education, is the notion of the journey, over time, as the student seeks to take on the mantle of scholarly teacher and leaderly teaching scholar. Future curriculum renewal should intentionally and sensitively be guided by these imperatives.

## Supplementary Information

**Additional file 1: Appendix 1. **Focus group prompts.

**Additional file 2: Appendix 2. **Interview prompts.

## Data Availability

The datasets used and analysed for the study are available from the corresponding author on reasonable request.

## Abbreviations

MHPE: Master’s in Health Professions Education
WFME: World Federation of Medical Education
