# Supplementary material for: Planned or emergent? An evaluation of a Master’s in Health Professions Education programme
Source: BMC Med Educ. 2022 Apr 4;22:242. doi: 10.1186/s12909-022-03319-5 (PMC8981925; doi:10.1186/s12909-022-03319-5)
Supplement: Supplementary file 1 — Additional file 1: Appendix 1. Focus group prompts. [file 12909_2022_3319_MOESM1_ESM.docx]

**Appendix 1: Focus group prompts**

The interviewer asked the students to reflect on their learning experiences during their first year in the programme. These prompts were based on transformative learning principles namely critical reflection on personal experiences.

Can you reflect on the various modules that you did this past year?

- reflect in terms of how engaging the various face to face and online presentations / interactions were
- and whether you feel that the specific module outcomes were achieved
- which factors enhanced your learning
- which factors constrained your learning
