# Supplementary material for: Planned or emergent? An evaluation of a Master’s in Health Professions Education programme
Source: BMC Med Educ. 2022 Apr 4;22:242. doi: 10.1186/s12909-022-03319-5 (PMC8981925; doi:10.1186/s12909-022-03319-5)
Supplement: Supplementary file 2 — Additional file 2: Appendix 2. Interview prompts. [file 12909_2022_3319_MOESM2_ESM.docx]

**Appendix 2: Interview prompts:**

The interviewer asked the students to reflect on their learning experiences during their second year and also the entire programme. These prompts were based on transformative learning principles namely critical reflection on personal experiences and different ways of doing.

1. What was your initial expectations when you applied for, and embarked on, your studies in the MPhil in HPE programme?
2. Have these expectations been met? (If yes) In what ways? (If not) Why not?
3. Please expand on how you see yourself as researcher, educational leader, scholar, academic writer, …, having completed the MPhil in HPE.
4. If you reflect back over time since you started your MPhil in HPE studies, how have your perceptions and use of the following changed? Please explain…..

- Teaching practice
- Adoption of an evidence-based approach
- Blended learning
- Inter-professional/ Collaborative practice
- Social accountability
- Being able to operate in a resource constrained environment
- Any other issues you would like to address?

5 Has your current environment afforded you any opportunities to add value now that your studies are completed? Have you faced any challenges?

6 Lastly, elaborate on the role of the longitudinal ePortfolio in enabling a transformative learning experience. (Disposition to reflective practice prompt if necessary)

7 Are there any other reflections that you would like to share with regard to your experience of being an M Phil in HPE student?

**Thank you for your time.**
